# Emergence of Longitudinal Queues in Group Navigation: An Interpretable Approach via Projective Simulation

**DOI:** 10.3390/biomimetics11030201

**Published:** 2026-03-10

**Authors:** Decheng Kong, Kai Xue, Ping Wang, Zeyu Xu

**Affiliations:** College of Mechanical and Electrical Engineering, Harbin Engineering University, Harbin 150001, China; kongdecheng@hrbeu.edu.cn (D.K.); xuekai@hrbeu.edu.cn (K.X.); xuzeyu@hrbeu.edu.cn (Z.X.)

**Keywords:** collective behavior, projective simulation, longitudinal queues, group navigation

## Abstract

The formation of longitudinal queues is critical for biological and artificial swarm systems to achieve efficient long-distance navigation. However, the “black-box” nature of conventional deep reinforcement learning models often obscures the microscopic rules driving the emergence of such ordered behaviors. To address this challenge, this paper proposes an interpretable computational model of collective behavior based on Projective Simulation and Episodic Compositional Memory, which enables individuals to learn decision-making strategies within a transparent state–action network. Simulation results demonstrate that the swarm can self-organize into stable and highly elongated longitudinal queues. Crucially, through visualization of microscopic strategies, we reveal a deterministic target-priority mechanism: when local neighbor alignment conflicts with global target orientation, individuals learn to strictly prioritize the target direction, serving as the key driving force for queue formation. Further parametric analysis indicates that the action space granularity exerts a nonlinear impact on stability, identifying moderate control precision as the optimal choice. This study not only provides a transparent computational explanation for the self-organization mechanism of queues in collective motion but also offers a theoretical foundation for designing interpretable swarm navigation systems.

## 1. Introduction

In the natural world, collective motion is one of the most remarkable and thought-provoking phenomena [1,2,3,4]. From microscopic bacteria colonies [5,6,7] to macroscopic animal groups [8,9,10,11], this coordinated behavior across dozens of orders of magnitude in space and time demonstrates the remarkable self-organization characteristics of living systems. This phenomenon is not a chaotic random aggregation but follows profound physical and biological laws. Understanding the microscopic individual interaction mechanisms behind these macroscopic ordered behaviors is not only crucial for biological research but also provides important inspiration for designing efficient and robust swarm robotic systems [12].

Notably, the morphology of collective motion often changes dynamically with task objectives. Particularly in long-distance migration or navigation tasks, many biological groups self-organize into highly sequential longitudinal queues [13,14,15]. For example, spiny lobsters form organized single-file lines during seabed migration to reduce fluid resistance [16,17]; army ants form traffic flows in complex terrain to avoid collisions and congestion [18,19]. This queue structure is believed to significantly enhance movement efficiency and navigation stability [17,20,21]. However, determining how this highly ordered linear formation emerges without central command, relying solely on local individual interactions (such as visual alignment), remains a challenge in collective behavior research and a key standard for verifying whether computational models can replicate real biological intelligence.

To explore the formation mechanisms of collective behavior, early research relied primarily on rule-based models, such as the classic Vicsek model [22] and Reynolds’ Boids model [23]. These models assumed individuals follow simple local rules (repulsion, alignment, and attraction) and successfully replicated many basic collective formations [24,25,26,27]. However, when facing specific tasks like long-distance navigation, environmental complexity and target-oriented requirements make it extremely difficult to manually design optimal local rules [28]. These traditional models often lack adaptability and struggle to simulate the process of organisms optimizing strategies through learning in dynamic environments.

In recent years, multi-agent reinforcement learning (MARL), especially deep reinforcement learning (DRL), has shown excellent performance in solving complex swarm collaboration tasks [29,30,31,32,33]. Although DRL can automatically acquire efficient strategies through interaction with the environment, its decision-making process based on deep neural networks is typically a black box [34]. It is difficult to parse exactly what interaction rules the individuals have learned or to explain their decision logic from a biological perspective. This lack of interpretability limits our ability to reverse-engineer biological cognitive mechanisms through computational models [35,36].

In response to these challenges, this study proposes an interpretable computational model of collective behavior based on Projective Simulation (PS) [37] and Episodic Compositional Memory (ECM). Unlike traditional deep learning black boxes, the PS model provides a transparent random-walk decision mechanism [38,39]. Its core ECM structure allows us to directly access and analyze the mapping between internal states and actions. This architecture not only grants individuals learning capabilities in long-distance navigation but, more importantly, enables us to “open the black box” and directly observe and extract the microscopic strategy rules driving the emergence of collective behavior. Furthermore, inspired by neuroscience research showing that the prefrontal cortex represents continuous movement directions in a discretized coding pattern, we introduce a biologically plausible discretized action space into the model [40]. Compared to the general PS framework for collective motion [39], our work focuses on the specific navigational challenge of queue formation and advances the methodology by providing a systematic way to interpret the internal logic of the learned *H*-matrix, revealing how cognitive priorities shift during navigation.

The primary contribution of this work to the advancement of biomimetics lies in the transition from mimicking behavior to mimicking decision mechanisms. Unlike traditional black-box models, our framework provides a transparent computational explanation for how individual learning leads to the self-organization of longitudinal queues. We identify a deterministic target-priority mechanism that explains how biological groups balance social alignment with global navigation. Furthermore, we provide evidence for the intermediate complexity hypothesis regarding action granularity, suggesting that a moderate level of control precision is required to balance maneuverability and learnability. Simultaneously, we demonstrate that even if perceptual information is compressed to a minimalist discrete state, it is sufficient to support complex long-distance navigation. These findings offer scalable and interpretable design principles for biomimetic swarm robotic systems.

The paper is organized as follows: an introduction to Projective Simulation and a detailed description of the model and the learning setup are given in Section 2. In Section 3, we analyze the macroscopic emergence of longitudinal queues and the microscopic target-priority mechanism, followed by performance benchmarks against Vicsek and DQN models. We also further examine parameter sensitivity and validate the model’s robustness and generalization across diverse environments. Section 4 summarizes the findings and concludes the paper.

## 2. Model and Evaluation Indicators

In this work, we employ Projective Simulation, a model of agency defined as the capacity of an autonomous entity to make independent decisions based on its internal state and environmental sensory input. Each individual is modeled as an artificial agent that perceives its surroundings, makes decisions, and performs actions within a closed-loop interaction. Within the PS model, individual decision-making is integrated into a reinforcement learning framework, allowing for the design of specific scenarios and tasks that individuals should solve, followed by the study of the resulting strategies developed by the individuals. While other RL algorithms can be used to model learning individuals, Projective Simulation is particularly suitable for modeling collective behavior because it provides a clear and transparent structure with direct access to the individual’s internal state. This allows the deliberation process to be analyzed in an explicit way and related to the individual’s behavior. Such analysis helps us gain new insights into how and why individual interactions leading to collective behavior emerge.

### 2.1. Projective Simulation Model

**Figure 1:**
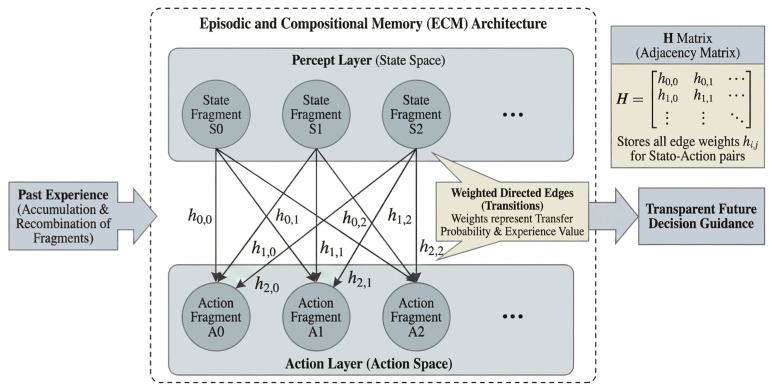
Schematic of the ECM architecture illustrating the transparency of the decision-making process. The model uses an adjacency *H*-matrix to store experience values between percept and action layers, enabling a direct readout of the learned rules as a probability table, which avoids the “black-box” nature of traditional deep learning.

ECM simulates the process of individuals guiding future decisions by accumulating and recombining past experience fragments, with its structure and operational mechanism fundamentally ensuring decision transparency. ECM employs a two-layer network structure, including a Percept Layer (State Space) and an Action Layer (Action Space). These layers are connected by weighted edges to form a complete decision graph, as shown in Figure 1. Mathematically, the ECM can be represented as a directed weighted graph, where each node corresponds to a clip (fragment) and each edge corresponds to a transition between two clips, with weights determining the transition probabilities. Percept-layer nodes correspond to the discretized state space, while action-layer nodes correspond to executable steering actions. All edge weights are stored in the adjacency matrix of the graph, termed the *H*-matrix, which records the experience value of different state–action pairs.

Individuals interact with the environment and make decisions via the ECM, with the core decision mechanism based on the probability calculation of state–action mappings. When an individual receives a sensory signal, the corresponding percept node is activated, starting a random walk to select an action node, which triggers a specific movement behavior. For a given percept state *s*, the probability of choosing action *a* is determined by the weights in the *H*-matrix:

(1) $$P(a|s)=\frac{h_{s,a}}{\sum_{k}h_{s,k}}$$

where $h_{s,a}$ is the weight from state *s* to action *a*, and the denominator is the normalization factor over all possible actions. This formula ensures that decisions are stochastic yet influenced by past experience.

The individual strategy learning process centers on shaping behavior probability distributions through a reward mechanism, relying on the synergy of two core matrices: the *H*-matrix and the *G*-matrix. The *H*-matrix acts as long-term memory, recording cumulative experience values:

(2) $$H(t+1)=H(t)+R_{total}\cdot G(t)$$

where $R_{total}$ is the environmental reward. The *G*-matrix serves as a short-term memory mechanism, marking recently activated behavioral paths through a glow effect. The element $g_{s,a}$ represents the glow value of the edge connecting percept *s* to action *a*. When a state–action pair is activated at time *t*, its corresponding glow value is updated as:

(3) $$g_{s,a}(t+1)=(1-\eta )g_{s,a}(t)+\delta_{s,a}$$

where η is the glow parameter determining the decay speed of short-term memory, and $\delta_{s,a}$ is an indicator function that is 1 if the state–action pair was activated and 0 otherwise. This mechanism allows reward signals to propagate backward along an action sequence.

The *H*-matrix, which serves as the individual’s long-term memory, is initialized at the beginning of the training process as a uniform matrix where all entries $h_{s,a}$ are set to 1. At the end of each training episode, the *H*-matrix state is fully preserved and used as the initial strategy for the next episode. In contrast, the *G*-matrix is initialized to 0 at the start of each episode to clear past records. Specifically, when a reward is obtained, the system distributes the signal via the *G*-matrix to all recently involved state–action edges. This allows individuals to reinforce not just the final rewarded action but the series of continuous decisions leading to success. Over repeated iterations, frequently rewarded paths gain higher weights in the *H*-matrix, significantly increasing their selection probability. Ultimately, the learned strategy is stored in the *H*-matrix; for any state *s*, the relative preference for actions can be clearly seen by viewing all values of $h_{s,a}$, which is equivalent to a readable state–action probability lookup table.

### 2.2. Details of the Proposed Model

This study considers a group of *N* PS individuals, each with an identical ECM structure and learning mechanism, sharing the same ECM matrix. This can be seen as a simplified model for species with low cognitive capacity or as a theoretical approach to study optimal behavior emerging under certain conditions. We set a long-distance navigation task: individuals must move to a distant target (simulating a food source) in an unbounded 2D space, deciding based solely on local neighbor directions and the global target orientation.

In this work, all individuals in the group share an identical ECM structure and the same *H*-matrix. This design choice is based on two considerations:

• Universality of Self-Organization: We aim to identify the fundamental interaction rules that allow a group to achieve ordered navigation without relying on individual specialization;
• Minimal Cognitive Load: We aim to demonstrate that a shared, low-complexity strategy based on a discretized state space is sufficient to drive the emergence of longitudinal queues.

While biological systems often exhibit heterogeneity, this shared-strategy baseline serves as a crucial reference for understanding the lower bound of cognitive requirements for collective order.

#### 2.2.1. State Space

Based on the neural mechanisms of biological visual systems, we designed a dual-dimension state space centered on local relative direction perception and global target navigation. The first dimension captures the relative direction between the individual and its neighbors, corresponding to neural encoding for local motion direction [41]. The second dimension represents the navigation relationship with the global target, simulating innate magneto-reception or visual landmark navigation [42].

**Figure 2:**
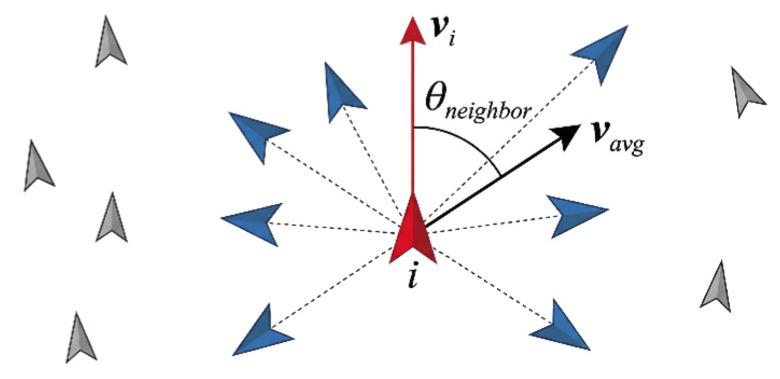
Geometric definition of the relative neighbor direction angle $\theta_{neighbor}$. This metric quantifies the angular deviation between the focal individual *i* and the average velocity vector of its topological neighbors, providing the local social cue for alignment.

The relative direction angle for the first dimension is calculated based on the geometric relationship between the individual’s direction and the average direction of its neighbors (Figure 2). For focal individual *i*, the set of neighbors $N_{i}$ is defined following a topological approach, where *i* interacts with its *k* nearest neighbors (set as k=6 in this study). This metric-free interaction ensures that each individual maintains social alignment regardless of local density, which is consistent with the coordination mechanisms observed in many biological groups [43]. The average movement direction is then calculated as:

(4) $$v_{avg}=\frac{1}{\left|N_{i}\right|}\sum_{j\in N_{i}}v_{j}$$

where $v_{j}$ is the velocity vector of neighbor *j*. The angle $\theta_{neighbor}$ between the individual’s direction and the neighbor average is:

(5) $$\theta_{neighbor}=\mathrm{sign}({\hat{v}}_{i}\times {\hat{v}}_{avg})\cdot \arccos({\hat{v}}_{i}\cdot {\hat{v}}_{avg})$$

where the unit vectors ${\hat{v}}_{i}$ and ${\hat{v}}_{avg}$ represent the movement direction of the focal individual *i* and the average movement direction of its neighbors, respectively, and sign(x) denotes the sign function. The value of this angle ranges from [−π,π], where a negative value indicates that the average direction of the neighbors is clockwise relative to the focal individual’s movement direction, while a positive value indicates it is counter-clockwise.

**Figure 3:**
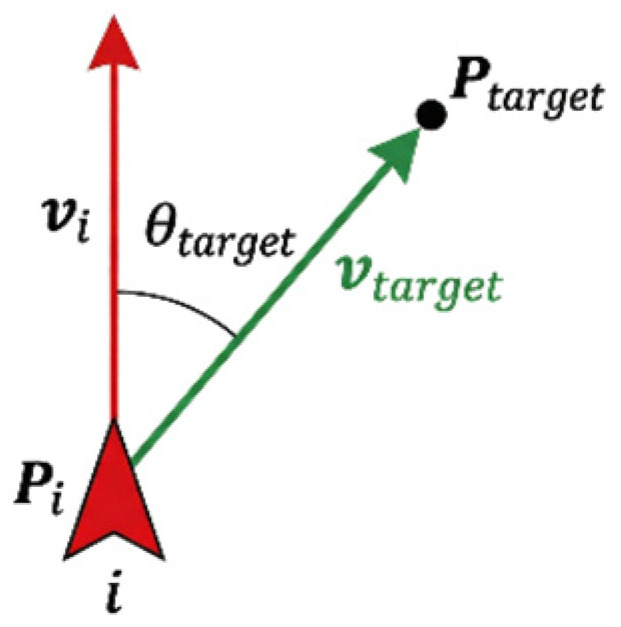
Geometric definition of the relative target angle $\theta_{target}$. This parameter measures the orientation of the focal individual *i* relative to the target, serving as the global navigational cue.

The relative direction angle of the second dimension characterizes the navigational relationship between the individual and the global target. This is obtained by calculating the angle between the individual’s current movement direction and the direction toward the target, as illustrated in Figure 3.

(6) $$v_{target}=P_{target}-P_{i}$$

(7) $$\theta_{target}=\mathrm{sign}({\hat{v}}_{target}\times {\hat{v}}_{i})\cdot \arccos({\hat{v}}_{target}\cdot {\hat{v}}_{i})$$

where $P_{target}$ and $P_{i}$ represent the positions of the target and the individual *i*, respectively. Similarly, the value of this angle ranges from [−π,π], where a negative value indicates that the target direction is clockwise relative to the focal individual’s movement direction, while a positive value indicates it is counter-clockwise.

**Figure 4:**
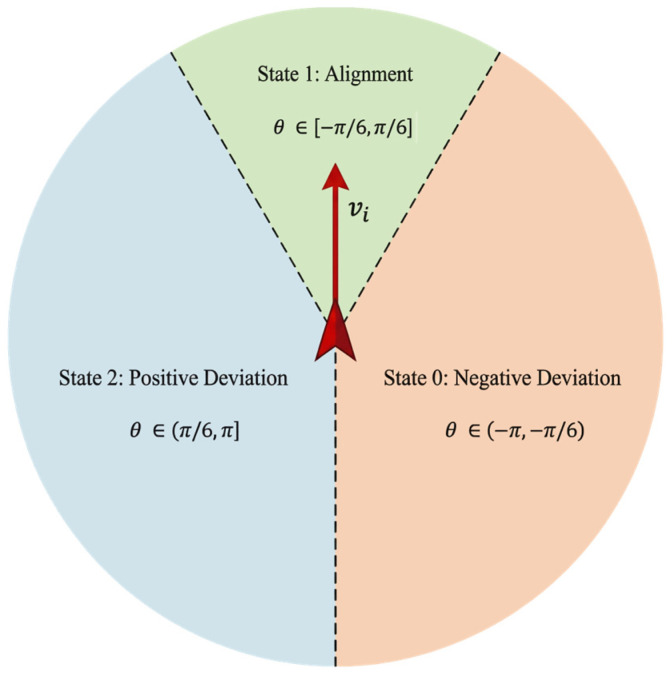
Discretization mapping of continuous sensory input into three semantic intervals. To simulate biological categorical perception, continuous angles are mapped to Negative Deviation, Alignment, and Positive Deviation, which reduces the state space to (3×3) states.

Furthermore, neuroscience research indicates that the processing of angular information in biological visual systems exhibits distinct categorical perception characteristics; specifically, the representation of continuous motion directions in the prefrontal cortex follows a typical discretized coding pattern [44]. Therefore, we adopt a unified discretization scheme for both the local social cue ($\theta_{neighbor}$) and the global navigational cue ($\theta_{target}$). The continuous relative direction angles of the two dimensions are discretized into three semantically clear intervals: negative deviation, alignment, and positive deviation, as illustrated in Figure 4. Such a division not only aligns with the information processing methods of biological neural systems but also streamlines the dimensions of the state space.

**Figure 5:**
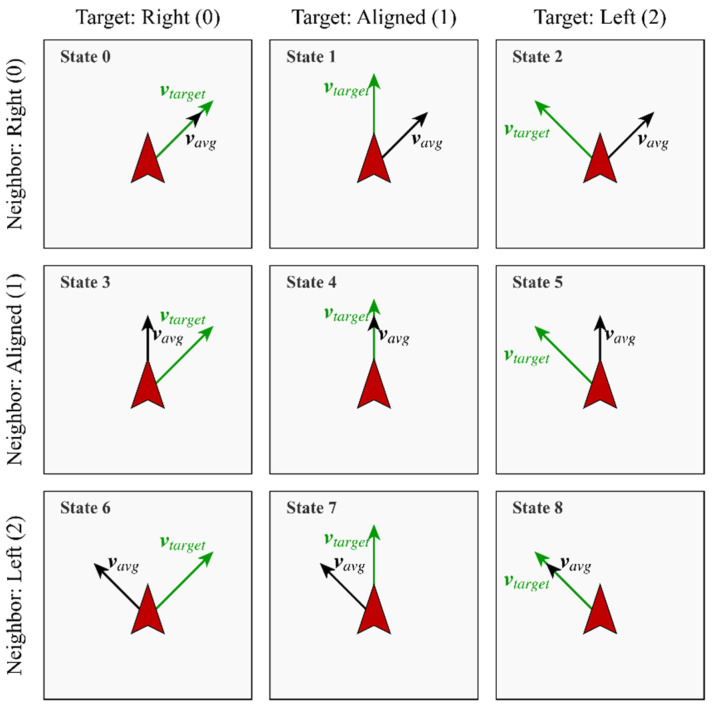
Visual encoding of the complete nine-state discrete percept space. Each state represents a unique combination of neighbor and target orientations, providing the basic reference for the individual to learn complex arbitration rules.

The discretization function is defined as follows:

(8) $$s_{n}=\left\{\begin{array}{ll} 1(Alignment), & \mathrm{if}\;|\theta |\leq \pi /6\\ 0(Right), & \mathrm{if}\;-\pi <\theta <-\pi /6\\ 2(Left), & \mathrm{if}\;\pi /6<\theta \leq \pi \end{array}\right.$$

when the relative angle θ<−π/6, it is defined as State 0, indicating that the neighbors’ average direction deviates to the right of the individual; when θ∈[−π/6,π/6], it is defined as State 1, indicating that the individual maintains basic alignment with the movement direction of the neighbors; and when θ>π/6, it is defined as State 2, indicating that the neighbors’ average direction deviates to the left of the individual. Therefore, the individual’s two-dimensional discretized state space can be expressed as:

(9) $$S=\left\{\left(s_{n},s_{t}\right)|s_{n}\in \left\{0,1,2\right\},s_{t}\in \left\{0,1,2\right\}\right\}$$

where $s_{n}$ and $s_{t}$ represent the sets of states for neighbor orientation and target orientation, respectively. Consequently, the complete state space is composed of the combination of these two dimensions, containing a total of 3×3=9 states, with the specific encoding shown in Figure 5.

#### 2.2.2. Action Space

Consistent with most collective behavior models [22,34], it is assumed that individuals maintain a constant linear velocity, and movement control is primarily achieved by adjusting angular velocity. The core idea of the action space is to discretize continuous steering movement into a finite number of basic actions, enabling individuals to achieve complex movement behaviors through simple action choices. Therefore, the action space consists of a series of discrete steering angles, adopting a design principle of symmetrical distribution.

**Figure 6:**
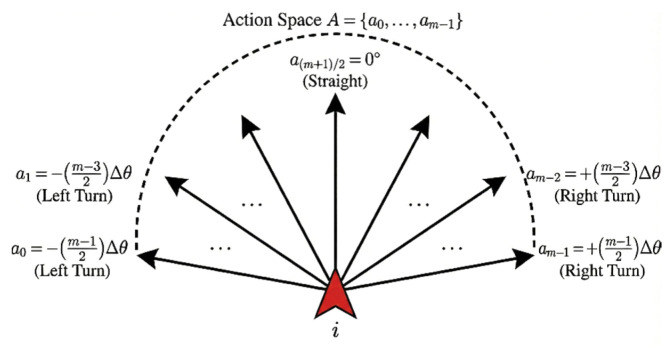
Distribution of the discretized action space with *m* steering options. The symmetrical layout includes a 0° straight action and multiple left/right turn units, allowing the individual to fine-tune its trajectory through reinforcement learning.

Assuming the total number of actions for an individual is *m* and the basic steering unit is Δθ, as shown in Figure 6, the action space set can be expressed as:

(10) $$A=\left\{a_{k}|k=0,1,2,\ldots ,m-1\right\}$$

where the number of actions is set to an odd integer to ensure the inclusion of a straight movement action while maintaining left–right symmetry. Each action $a_{k}$ corresponds to a specific steering angle, and its mathematical expression is:

(11) $$a_{k}=(k-\frac{m-1}{2})\cdot \Delta \theta$$

where the angle of zero corresponds to a straight-line movement, while a sequence of negative angles corresponds to right-turn actions, and a sequence of positive angles corresponds to left-turn actions. Consequently, the maximum steering angle for an individual is:

(12) $$\theta_{\max}=\pm \frac{m-1}{2}\cdot \Delta \theta$$

For any selected action $a_{k}$, the update of the individual’s movement direction is implemented by a standard rotation matrix:

(13) $$v_{i}(t+1)=\left[\begin{array}{cc} \cos\;\alpha_{k} & -\sin\;\alpha_{k}\\ \sin\;\alpha_{k} & \cos\;\alpha_{k} \end{array}\right]\cdot v_{i}(t)$$

#### 2.2.3. Reward Function

The reward function provides behavioral evaluation criteria for individuals through environmental feedback mechanisms, thereby driving the optimization and improvement of strategies, and plays a crucial role in guiding individual learning. This model adopts a composite reward function structure that comprehensively considers the multiple objectives of navigation efficiency and swarm coordination. Specifically, a composite reward function based on target navigation is designed, which consists of two parts: distance-change reward and directional consistency reward.

The distance-change reward focuses on evaluating changes in the proximity between the individual and the target point. Its core concept is to reward behaviors that effectively shorten the distance to the target:

(14) $$R_{dis}=(d(t-1)-d(t))$$

where d(t−1) and d(t) represent the distance between the individual and the target point at adjacent time steps. When an individual performs an action that results in a decrease in distance to the target, a positive reward is granted; conversely, when the distance increases, a corresponding penalty is applied.

The directional consistency reward focuses on the degree of matching between the individual’s movement direction and the target direction. By calculating the dot product of the individual’s current velocity direction and the target direction vector, the consistency between the two is evaluated:

(15) $$R_{dir}=\max(0,{\hat{v}}_{i}\cdot {\hat{T}}_{i})$$

when the individual’s movement direction is consistent with the target direction, a higher orientation reward is granted; as the direction deviates, the reward decreases accordingly. This design ensures that individuals not only approach the target point in terms of position but also maintain the correct heading in their movement posture, thereby achieving a more efficient and stable navigation process.

Ultimately, the composite reward function integrates each reward component through a weighted sum:

(16) $$R_{total}=\lambda_{d}\cdot R_{dis}+\lambda_{r}\cdot R_{dir}$$

where $\lambda_{d}$ is the distance reward coefficient and $\lambda_{r}$ is the orientation reward coefficient.

It is important to emphasize that the composite reward function $R_{total}$ is designed to represent the fundamental biological drive for efficient navigation rather than to prescribe specific formation rules. The reward signals solely evaluate the individual’s progress toward the target and their heading consistency. Crucially, no explicit rewards or penalties are associated with collective formations such as queuing or alignment. Therefore, the ordered macro-structures observed in the simulations are emergent behaviors arising from the reinforcement of micro-strategies that optimize these basic navigational objectives.

### 2.3. Evaluation Indicators

To comprehensively evaluate model performance, we define evaluation indicators at both the microscopic strategy and macroscopic behavior levels. Microscopic strategy indicators are used to quantify the certainty, randomness, and interpretability of individual decisions, including the ECM probability table, average entropy [45], and average certainty. Macroscopic behavior indicators are used to quantify the structural properties, coordination, and morphological characteristics of the collective motion, including the polarization [22] and elongation ratio.

**ECM Probability Table:** After training, the complete probability distribution matrix is extracted from the *H*-matrix according to the normalization rule in Equation (1). The matrix element $P_{ij}$ represents the probability of selecting the *j*-th action under the *i*-th percept state.

**Average Entropy:** Entropy is a concept in information theory for measuring the uncertainty of random variables. In the analysis of reinforcement learning strategies, it can effectively measure the degree of decision-making certainty of a strategy under different states. For each percept state *s*, the entropy of its strategy distribution is calculated as:

(17) $$H(s)=-\sum_{a\in A}P(a|s)\cdot \log_{2}P(a|s)$$

where P(a|s) is the action probability distribution obtained from the ECM probability table. The average entropy is then defined as the average value of the entropy for all states:

(18) $$\bar{H}=\frac{1}{\left|S\right|}\sum_{s\in S}H(s)$$

unlike an ensemble average, this state-wise average treats each of the 9 perceptual configurations as equally important. This allows us to evaluate the convergence of the policy even in conflict states. Therefore, a lower average entropy indicates higher strategy certainty, meaning the individual has clear preferred actions in most states; conversely, a higher average entropy indicates higher strategy randomness, where the individual retains explorative tendencies in more states and tends to make balanced choices among various possible actions.

**Average Certainty:** Defined as the average of the maximum probabilities across the 9 percept states, its calculation formula is as follows:

(19) $${\bar{P}}_{\max}=\frac{1}{\left|S\right|}\sum_{s\in S}\underset{a\in A}{\max}P(a|s)$$

Consequently, a higher average certainty indicates a higher degree of strategy convergence, meaning the individual has clear action preferences in most states.

**Polarization:** The polarization $O_{p}$ is currently one of the most commonly used evaluation metrics for collective movement behavior. By quantifying the degree of consistency in the movement directions of individuals within a group, it intuitively demonstrates the concentration of the group in its overall orientation. Its definition is as follows:

(20) $$O_{p}=\frac{1}{N}\|\sum_{i=1}^{N}\frac{v_{i}}{\left\|v_{i}\right\|}\|$$

where *N* represents the group size, and $v_{i}$ is the movement velocity of individual *i*. The polarization $O_{p}$ ranges from [0,1]. When the group is in an ordered state—meaning all individuals move in substantially the same direction—$O_{p}\approx 1$. When the group is in a disordered state—where individual movement directions are completely random—$O_{p}\approx 0$. Therefore, a higher value of $O_{p}$ indicates that the movement directions of individuals are more aligned, representing a higher degree of order in the collective motion.

**Elongation Ratio:** To quantify the morphological evolution and longitudinal sequentiality of the group, we define the elongation ratio $O_{e}$. At each time step, a Minimum Bounding Box (MBB) is constructed to enclose the entire group, with one axis strictly aligned with the group’s current centroid movement direction. The ratio is defined as:

(21) $$O_{e}=\frac{L}{W}$$

where L and W represent the length along and the width perpendicular to the direction of motion, respectively. A value of $O_{e}>1$ indicates that the group is slender and stretched along the direction of travel, serving as a key morphological indicator for the emergence of longitudinal queues.

## 3. Numerical Simulation and Results

To verify the effectiveness of the proposed model in collective navigation tasks and explore the microscopic mechanisms of queue emergence, we conducted large-scale numerical simulations in a two-dimensional unbounded space. The experimental process consists of two stages: the policy training stage and the model evaluation stage. This section first describes the detailed experimental settings, then analyzes the evolution of the group’s macroscopic behavior based on the maturely trained model, reveals the interpretability of the strategy through the visualization of decision matrices, and finally discusses the non-monotonous influence of action space granularity on system stability.

### 3.1. Simulation Setup

The experiment simulates an open natural environment to avoid artificial constraints on collective behavior caused by boundary effects. The group consists of *N* individuals with the task of navigating to a distant static target. To obtain statistically significant results, we conducted 30 independent training runs. Each training run consists of 5000 episodes, with the maximum simulation duration for each episode set to 10,000 time steps.

At the beginning of each episode, individuals are randomly distributed within a radius of 300 units. Crucially, the initial orientation of each individual is independently and uniformly sampled from the interval [0,2π], ensuring no pre-existing alignment bias before the learning process begins, and the speed is 1. This setup ensures that there is no pre-existing alignment bias or broken symmetry in the collective heading before the learning process begins, thereby confirming that the subsequently observed longitudinal queues are an emergent property of the learned decision-making rules rather than an artifact of initial conditions. The *H*-matrix (long-term memory) is preserved and continues to accumulate experience. All experimental result data were obtained during the model evaluation stage—specifically, by loading the converged *H*-matrix after training, freezing policy updates, and performing simulation runs again to test performance. The core parameter settings of the model are shown in Table 1.

### 3.2. Emergence and Micro-Mechanisms of Collective Queue Formation

#### 3.2.1. Macroscopic Emergence of Longitudinal Queues

**Figure 7:**
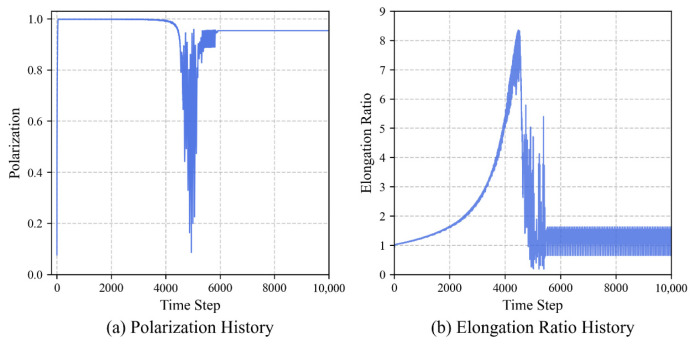
Temporal evolution of group order and morphology during the navigation task. (**a**) Polarization rapidly converges to 0.99 within 100 steps. (**b**) The elongation ratio climbs to a peak of 8.31 during steady-state navigation, quantitatively confirming the emergence of a slender longitudinal queue.

**Figure 8:**
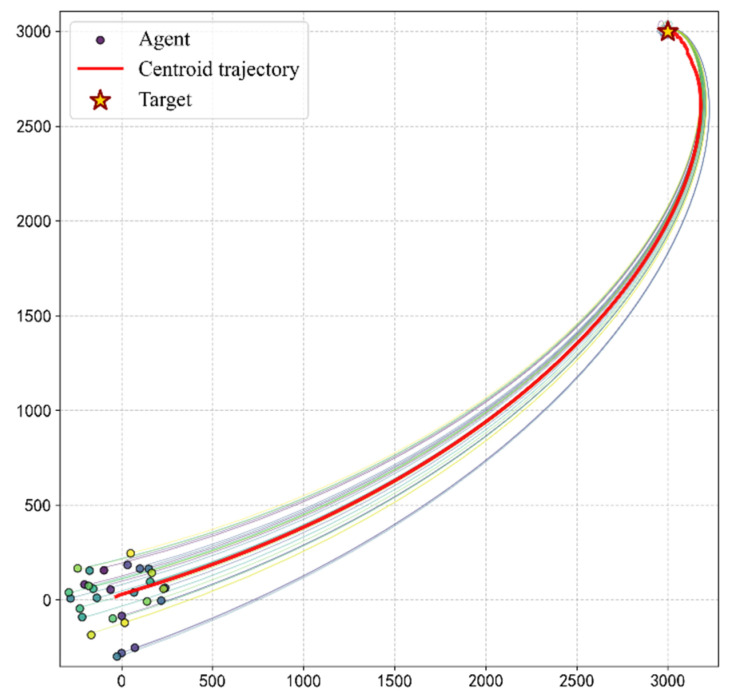
Visualization of efficient navigation trajectories toward the target. The red centroid trajectory shows a smooth, curved path, while individual paths demonstrate the transition from disordered initial positions to a highly aligned and stable sequential structure.

**Figure 9:**
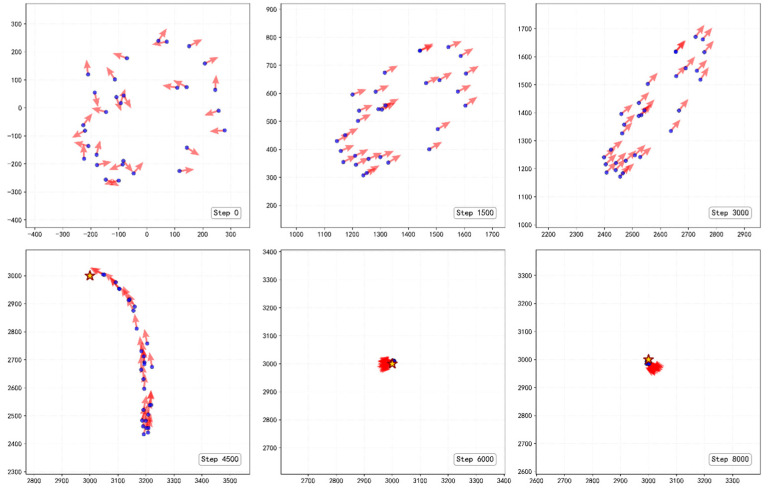
Snapshots of morphological transitions across different task stages. The group evolves from a random cluster (t=0) to a longitudinal queue during transit (t=1500,3000) and finally into a stable vortex structure surrounding the target (t=6000,8000).

Simulation evaluation results demonstrate that the fully trained group can self-organize into a stable longitudinal queue structure. As shown in Figure 7, Figure 8 and Figure 9, the evolution process of the group exhibits three typical stage characteristics:

**Rapid Alignment Stage (*t* < 100):** In the initial stage of the task, despite the random starting positions, the group quickly converges to a highly ordered state by utilizing the learned strategies. The polarization $O_{p}$ jumps from an initial 0.1 to 0.99 within just 100 steps, indicating that individuals can rapidly reach a collective consensus using local information.

**Steady-State Navigation Stage (100 < *t* < 4200):** The group advances toward the target while maintaining an extremely high degree of alignment. Notably, the elongation ratio in the evaluation data gradually climbs and reaches a peak ($O_{e}\approx 8.31$) during this stage. This indicates that the group is significantly stretched morphologically, forming a typical queue structure. This longitudinal queue structure effectively reduces lateral conflicts and enhances the efficiency of collective navigation. The smooth, curved morphology of the centroid trajectory (Figure 8) reflects the group’s dynamic adaptation during navigation. The discrete nature of the steering actions introduces a form of effective inertia, preventing the group from making instantaneous turns, thereby forming the observed pursuit curve, balancing directional alignment with the emergent structural stability of the queue.

**Target Adjustment Stage (*t* > 4200):** When the leading individuals reach the target area (approximately 4200 steps), the group must undergo sharp directional adjustments due to the static nature of the target point, leading to brief fluctuations in the polarization. After approximately 5500 steps, the group stabilizes again, and the behavioral pattern shifts from a translational queue to a rotational behavior surrounding the target, with the polarization stabilizing at around 0.98. This morphological transition reflects the robust dynamic adaptability of the trained model to environmental changes.

#### 3.2.2. Microscopic Decision Logic: The Target-Priority Mechanism

**Figure 10:**
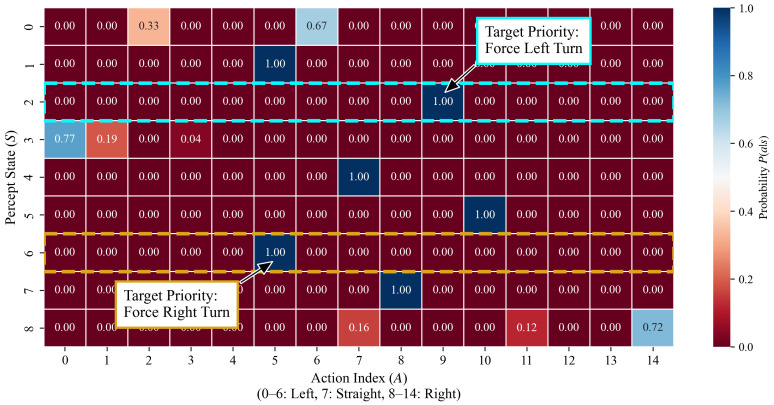
Visualization of the learned ECM probability matrix. The heatmap reveals high decision certainty (P(a|s)≈1) in 6 out of 9 states. Dashed boxes highlight conflict states (State 2 and State 6), where the individual learns to ignore neighbor alignment to strictly prioritize the target direction, providing the evidence target-priority. The numerical asymmetry is the result of spontaneous symmetry breaking during the reinforcement learning process.

A primary advantage of this model lies in its interpretability. By extracting and analyzing the trained ECM probability matrix (the normalized result of the *H*-matrix), we can directly observe the behavioral logic of individuals under different perceptual states. Figure 10 presents the action probability heatmaps for core states, revealing three key microscopic mechanisms for queue formation:

**High Decision Certainty:** As shown in Figure 10, after 5000 training episodes, the policy for 6 out of the 9 possible perceptual states converged to deterministic decisions (i.e., the selection probability for a specific action reached P(a|s)≈1). This low-entropy policy distribution establishes a low-noise communication channel within the group, enabling the steering signals of leading individuals to be accurately transmitted backward through simple local interactions.

**Target-Priority Conflict Arbitration Mechanism:** When the demand for local alignment conflicts with the requirements for global target navigation, the trained individuals demonstrate a clear hierarchy of priorities.

• Conflict Scenario 1 (State 2): Neighbors are on the right (requiring a right turn for alignment), but the target is on the left (requiring a left turn toward the target). The strategy matrix indicates that the individual selects Action 9 (a 4° left turn) with a probability of 1, prioritizing the target orientation;
• Conflict Scenario 2 (State 6): Neighbors are on the left (requiring a left turn for alignment), but the target is on the right (requiring a right turn toward the target). The individual primarily selects Action 5 (a 4° right turn) with a probability of 1, similarly prioritizing the target direction.

This target-priority arbitration rule breaks the equilibrium of pure aggregation and provides the group with a continuous forward driving force.

**Figure 11:**
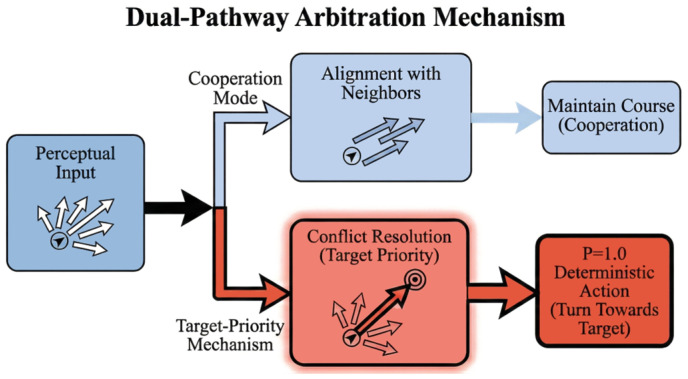
The Dual-Pathway Arbitration Mechanism: A hierarchical decision flow explaining queue emergence. The diagram illustrates how an individual processes perceptual input: (1) Under non-conflict conditions, it enters Cooperation Mode to maintain alignment with neighbors. (2) When a conflict arises between social alignment and the global goal, the individual activates the Conflict Resolution pathway, prioritizing the target direction (P(a|s)≈1). This prioritized switching breaks local symmetry and facilitates the formation of stable longitudinal queues.

**Deterministic Rule Extraction:** Based on the aforementioned probability analysis, we can extract the decision logic flow as shown in Figure 11. This logic reveals a clear Dual-Pathway Arbitration Mechanism: under normal, non-conflict conditions, individuals follow a Cooperation Mode, primarily maintaining alignment with their neighbors; however, once a conflict between local alignment and global target is perceived, the system immediately activates the Conflict Resolution pathway to execute the deterministic target-oriented priority strategy. This hierarchical dual-channel processing mode proves that combinations of simple local rules are sufficient to give rise to complex macroscopic ordered behaviors.

From a biomimetic perspective, the extracted target-priority rule (as shown in Figure 11) offers a novel perspective on the trade-off strategy in animal groups. While existing models often use linear weighting for multiple stimuli, our results suggest that biological individuals may prioritize navigation targets over social alignment under specific spatial constraints.

### 3.3. Comparative Analysis

#### 3.3.1. Comparison with Rule-Based Benchmark Models

**Figure 12:**
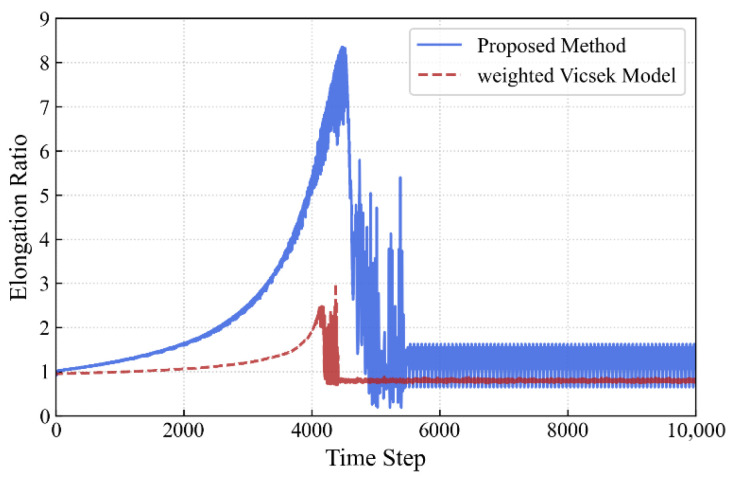
Comparison of elongation ratios between the PS model and the weighted Vicsek baseline. While the Vicsek model guides the group to the target, it remains a compact cluster ($O_{e}\approx 1∼2.5$) because its fixed linear rules cannot emulate the decisive target-priority arbitration of the PS model.

**Figure 13:**
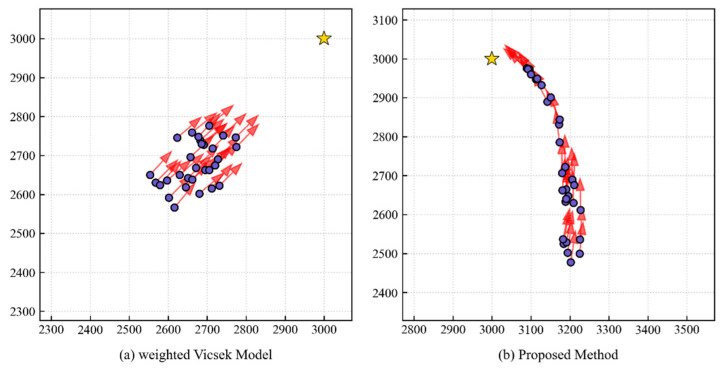
Morphological snapshots comparing rule-based and agency-based groups. (**a**) The weighted Vicsek model exhibits a dense aggregation. (**b**) The proposed PS method results in a distinct, elongated queue, highlighting the advantage of state-dependent learning strategies.

To further verify the superiority of the proposed PS model in the self-organized emergence of longitudinal queue structures, we conducted a comparative experiment between this model and the classic Vicsek model. Since the original Vicsek model only includes alignment rules, we introduced a target attraction term based on it to construct a weighted Vicsek Model as the baseline. In this baseline model, the movement direction $\theta_{i}(t+1)$ of individual *i* at time t+1 is determined by the linear weighting of the neighbors’ average direction $v_{avg}$ and the target direction $v_{target}$. The movement direction $\theta_{i}$ of individual *i* is updated synchronously at each time step and defined as:

(22) $$\theta_{i}(t+1)=\arg\left((1-\omega )\cdot \frac{v_{avg}}{\left||v_{avg}|\right|}+\omega \cdot \frac{v_{target}}{\left||v_{target}|\right|}\right)+\xi$$

where ω=0.5 is the target weight parameter used to adjust the individual’s level of focus on the global target, and ξ represents the random noise sampled from a uniform distribution [−0.25,0.25]. The comparison results are shown in Figure 12 and Figure 13. Experimental results indicate that although the weighted Vicsek model successfully guides the group to the target, the group exhibits a compact cluster morphology rather than the longitudinal queue formation characteristic of the PS model. Quantitative data shows that the elongation ratio of the Vicsek model remains at a low level ($O_{e}\approx 1∼2.5$), significantly lower than the peak reached by the PS model during the steady-state phase ($O_{e}\approx 8.31$).

This difference highlights the advantage of the PS model: the Vicsek model employs a fixed linear weighting rule, mechanically balancing the alignment and target vectors at all times, which prevents individuals from making decisive trade-offs between following neighbors and heading toward the target. In contrast, the ECM-based individuals learned a non-linear, state-dependent strategy. As described in Section 3.2, PS individuals are able to dynamically suppress alignment tendencies through the target-priority mechanism in critical conflict states (such as when the neighbors’ direction deviates from the target direction). This ability to flexibly switch strategies across different contexts is precisely the key mechanism for the emergence of efficient longitudinal queues, which is difficult for traditional simple-rule models to replicate.

While recent numerical work shows that line-like structures can emerge from non-reciprocal mechanical forces [46], our agency-based approach suggests a distinct cognitive pathway. In biological systems, queuing may arise not only from physical constraints but also from internal decision-making arbitration. Our findings reveal that individuals can learn to selectively decouple from social influence to prioritize navigational accuracy, suggesting that nature’s collective patterns may be driven by dual layers: one mechanical and one cognitive.

#### 3.3.2. Comparison of Performance and Interpretability with Deep Reinforcement Learning (DQN)

To further evaluate the advantages of the proposed PS model in terms of learning efficiency and policy transparency, we compared our model with a mainstream deep reinforcement learning algorithm—the Deep Q-Network (DQN) [28,29]. The DQN model employs a neural network containing two fully connected layers (each with 64 neurons and ReLU activation functions) to approximate the Q-value function. The input and output spaces are consistent with the PS model (input consists of 9 states with one-hot encoding, and the output consists of 15 discrete actions), and the training utilizes Experience Replay [47] (a technique that stores past experiences in a buffer to break data correlation) and Target Network [48] (a secondary network used to stabilize the learning process) mechanisms. These components are standard in Deep Q-Networks to ensure robust convergence but contribute to the “black-box” nature of the model compared to our transparent PS approach.

**Figure 14:**
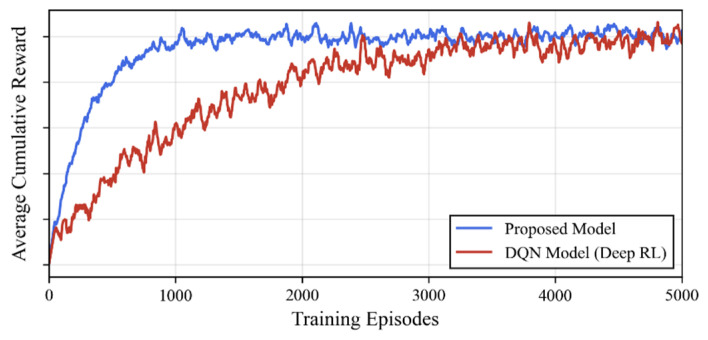
Comparison of learning efficiency between PS and DQN models. The PS model (blue) achieves faster convergence to maximum rewards than the DQN (red) and requires significantly fewer parameters, demonstrating its suitability for resource-constrained biomimetic systems.

The comparison results are shown in Table 2 and Figure 14. The experimental results reveal key trade-offs between the two learning paradigms:

**Comparable Navigation Performance but Different Learning Efficiencies:** As shown in Figure 14, both the DQN and PS models achieved a 100% navigation success rate, and their final group elongation ratios were similar (DQN: 8.1 vs. PS: 8.3), proving that both can learn efficient queuing strategies. However, in terms of convergence speed, because the PS model is based on direct tabular updates, its convergence speed is slightly faster than that of the DQN network, which requires gradient descent optimization.

**The Fundamental Difference Between Black Box and White Box:** This is the most critical distinction of this study. Although DQN can learn similar behaviors, its policy is encoded within thousands of weight parameters of the neural network, constituting a typical “black box”. We cannot directly interpret why DQN selects a specific action in a particular state, nor can we plot a clear logic flow diagram as shown in Section 3.2. In contrast, the ECM matrix of the PS model provides a completely transparent probability mapping, enabling researchers to directly read and verify microscopic rules such as the target-priority mechanism.

**Computational Resource Consumption:** The PS model does not require complex backpropagation calculations. Its parameter count consists of only 9×15=135 floating-point numbers, whereas the parameter count of even the lightweight DQN used in this experiment exceeds 5000. This means the PS model has higher deployment feasibility on hardware for computationally constrained swarm robotics.

In summary, while maintaining task performance comparable to Deep RL, the PS model offers unmatched advantages in interpretability and lightweight design, which are crucial for understanding the emergence mechanisms of collective behavior and ensuring system safety.

### 3.4. Parameter Sensitivity Analysis

To verify the robustness of the emergent collective behavior and ensure that the target-priority mechanism is not an artifact of specific parameter choices, we conducted a dual-dimension sensitivity analysis.

#### 3.4.1. Influence of Action Granularity

**Figure 15:**
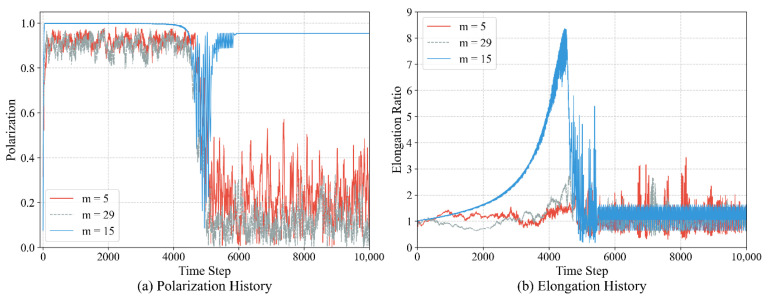
Non-monotonous impact of action granularity on group stability. Results show that moderate granularity (m=15) yields the highest polarization and elongation.

**Figure 16:**
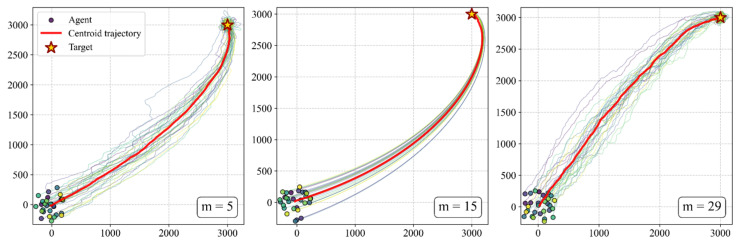
Trajectory patterns under different control precisions. Comparison shows that moderate granularity (m=15) produces the smoothest paths and most stable queue structures.

The discretization granularity is jointly determined by the number of actions (*m*) and the basic steering unit (Δθ), directly affecting the precision of individual steering control and the capability for strategy expression. In this experiment, under the condition of a fixed maximum steering angle $\theta_{\max}=14{}^{\circ}$, we explore the impact of different action counts on the formation and maintenance of the queue structure. Combined with the quantitative data in Table 3 and the visual analysis in Figure 15 and Figure 16, the experimental results demonstrate a significant non-monotonous relationship between granularity and queue quality:

**CoarseGranularity (m=5,Δθ=7°):** Due to the excessively large steering angles, individual movements appear rigid, making it difficult to perform subtle directional fine-tuning. This results in an average group alignment of only 0.56. Furthermore, due to frequent overshooting, the group is unable to form a compact structure, with a maximum elongation ratio of only 3.43.

**Fine Granularity (m=29,Δθ=1°):** Although the control precision is extremely high, the exponential expansion of the action space leads to a curse of dimensionality. The individuals struggle to explore the optimal strategy within 5000 training episodes, manifested as an extremely high strategy entropy (2.22) and decisions filled with random noise. Macroscopically, this causes the group to be disorganized internally and unable to maintain a queue, despite moving toward the target.

**Moderate Granularity (m=15,Δθ=2°):** This configuration achieves the optimal balance between control precision and learning efficiency. The individuals can quickly converge to a low-entropy strategy (average entropy of 0.44) while achieving smooth trajectory control. At this point, the group exhibits the highest alignment (0.95) and the largest longitudinal elongation ratio (7.92), proving that moderate control granularity is a key condition for the emergence of high-quality queue structures.

In summary, the discretization granularity is the decisive parameter for the precision of the queue structure. A moderate granularity (set at m=15 in this study) provides the group with behavioral primitives that are neither coarse nor redundant, serving as a necessary condition for generating stable, stretched, and adaptable queue structures. Coarse granularity prevents queue formation, while excessively fine granularity disrupts queue consistency due to learning noise. This finding indicates that moderate individual capability is a prerequisite for optimal collective intelligence.

Furthermore, the discretization of the action space introduces a fundamental effect of effective inertia. Due to the granulation of steering units (Δθ), individuals inevitably experience angular oscillations when aligning with targets, triggering persistent correctional maneuvers that manifest as curved trajectories. Our analysis shows that a moderate granularity (m=15) provides the balanced inertia necessary to stabilize longitudinal queues without the disordered oscillations seen at coarser scales. The specific handedness of these paths is an emergent result of stochastic symmetry breaking, with left- and right-handed curves occurring with equal probability. Ultimately, this smooth morphology represents a sophisticated trade-off between target-priority arbitration and action-induced inertia, mimicking the energy-efficient pursuit paths observed in biological migratory groups.

#### 3.4.2. Sensitivity Analysis of Reward Weights

**Figure 17:**
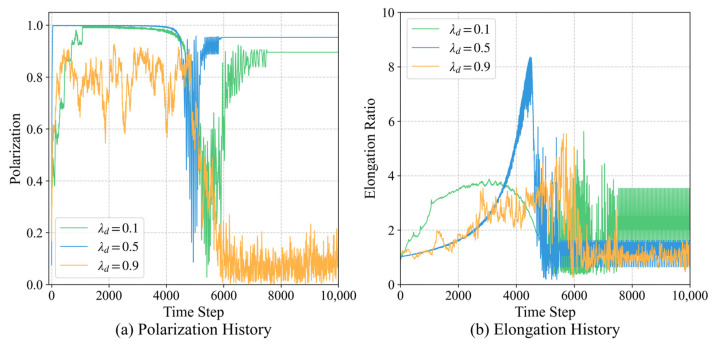
Impact of composite reward weights on collective navigation stability. (**a**) Temporal evolution of polarization under varying distance ($\lambda_{d}$) and orientation ($\lambda_{r}$) reward coefficients. (**b**) History of the elongation ratio, highlighting the optimal performance achieved in the balanced reward regime ($\lambda_{d}=0.5$, $\lambda_{r}=0.5$).

Beyond control precision, we investigate the influence of the composite reward weights, which balance the two primary navigational goals: shortening the distance to the target ($\lambda_{d}$) and maintaining directional consistency ($\lambda_{r}$). The results of the parameter sweep are summarized in Table 4 and Figure 17:

**BalancedRegime ($\lambda_{d}=0.5$, $\lambda_{r}=0.5$):** This configuration represents a Pareto-optimal solution where polarization and elongation are maximized simultaneously.

**Distance-Dominant Regime ($\lambda_{d}=0.9$):** As individuals prioritize proximity over postural alignment, the system experiences a sharp decline in order. At $\lambda_{d}=0.9$, polarization drops to 0.46, indicating that pure distance-pursuance without directional coordination leads to the collapse of the organized queue.

**Arbitration Robustness:** Crucially, across all tested weight combinations where successful navigation occurs, the target-priority mechanism remains the dominant strategy in conflict states. This confirms that prioritizing the global goal over local social cues is an intrinsic requirement for long-distance group navigation, rather than a result of arbitrary weight selection.

### 3.5. Robustness, Generalization, and Scalability Analysis

While the preceding sections confirm the emergence and interpretability of longitudinal queues in idealized environments, evaluating the model’s reliability under realistic constraints is essential to ensure its biological plausibility and robotic applicability. This section subjects the learned PS-based strategies to a series of comprehensive stress tests. Specifically, we examine the group’s performance across four critical dimensions: stochastic sensory noise, dynamic target tracking, coordinate independence, and group-size scalability. These analyses aim to validate that the deterministic target-priority mechanism functions as a stable coordination principle, resilient to external disturbances and operational scale-up.

#### 3.5.1. Robustness Under Environmental Noise

To evaluate the applicability of the proposed model in real-world biological and robotic contexts, we conducted a robustness analysis by introducing sensory noise. In natural environments, individual perception is inevitably subject to stochastic disturbances. We model the noisy perception of the relative neighbor angle $\theta_{neighbor}$ and target angle $\theta_{target}$ as:

(23) $${\theta^{\prime }}_{s}=\theta_{s}+\eta_{s},\;\eta_{s}\sim N(0,\sigma^{2})\;$$

where σ represents the sensory noise intensity.

**Figure 18:**
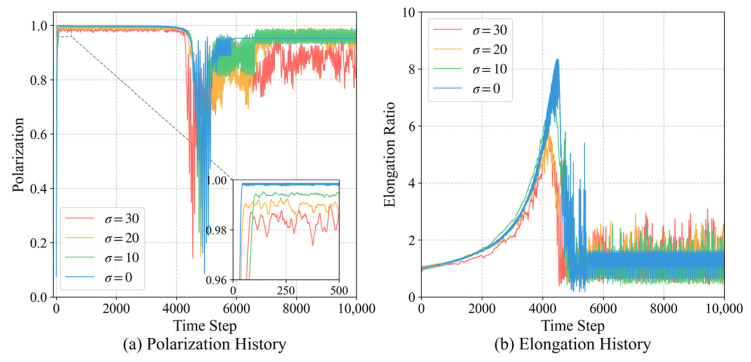
Robustness of collective order under varying sensory noise intensities. The group maintains high polarization (>0.97) even at σ=30°, proving that the learned target-priority mechanism and coarse-grained states effectively filter environmental disturbances.

Figure 18 illustrates the temporal evolution of group metrics under varying noise intensities (σ=0°,10°,20°,30°). As shown in Figure 18a, the group maintains a high polarization ($O_{p}>0.97$) even when sensory noise reaches 30°, indicating that the learned target-priority mechanism provides a strong stabilizing force for collective alignment. However, as noise increases, the fluctuations in polarization become more pronounced, and the time required for initial alignment increases.

The impact of noise on the elongation ratio is shown in Figure 18b. While the peak elongation ratio decreases with increasing noise intensity, the group is still capable of forming distinct longitudinal queues ($O_{e}>5$).

**Figure 19:**
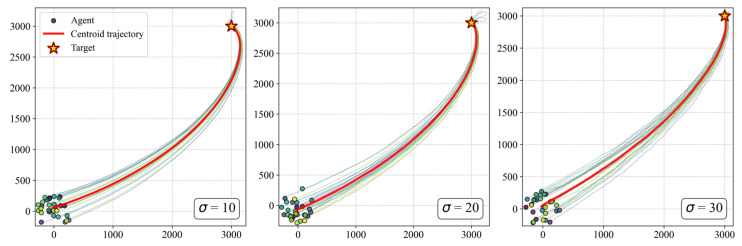
Visual comparison of collective trajectories under varying sensory noise intensities (σ=10°, 20°, 30°). The snapshots demonstrate that the group remains capable of navigating toward the target even when individual perceptions are subject to significant stochastic disturbances.

To visually complement the quantitative analysis, Figure 19 illustrates the collective trajectories under different noise intensities (σ=10°,20°,30°). The snapshots reveal that while higher noise levels introduce more frequent local path fluctuations, the overall group integrity remains intact. This visual evidence confirms that the target-priority mechanism acts as a robust directional filter, allowing the group to suppress individual sensory errors and maintain an effective navigational heading even in highly stochastic environments.

These results demonstrate that the ECM-based decision-making strategy possesses an inherent error-correcting capacity. The coarse-grained state perception effectively filters out low-amplitude noise, allowing the core navigation strategy to remain robust in non-idealized environments. This confirms that the minimalist cognitive mechanism proposed in this study is sufficient for maintaining complex spatiotemporal order under realistic constraints.

#### 3.5.2. Generalization to Dynamic Target Tracking

To evaluate the generalizability of the trained PS model, we extended the navigation task to a dynamic scenario where the target moves at a constant velocity $v_{target}=(-0.35,0.35)$, simulating the pursuit of a moving food source or a changing migration goal. The velocity vector (−0.35,0.35) is strategically chosen to maintain a biologically plausible speed ratio (ensuring target reachability) and to force continuous variations in the relative target bearing ($\theta_{target}$), thereby validating the model’s generalization in dynamic environments.

**Figure 20:**
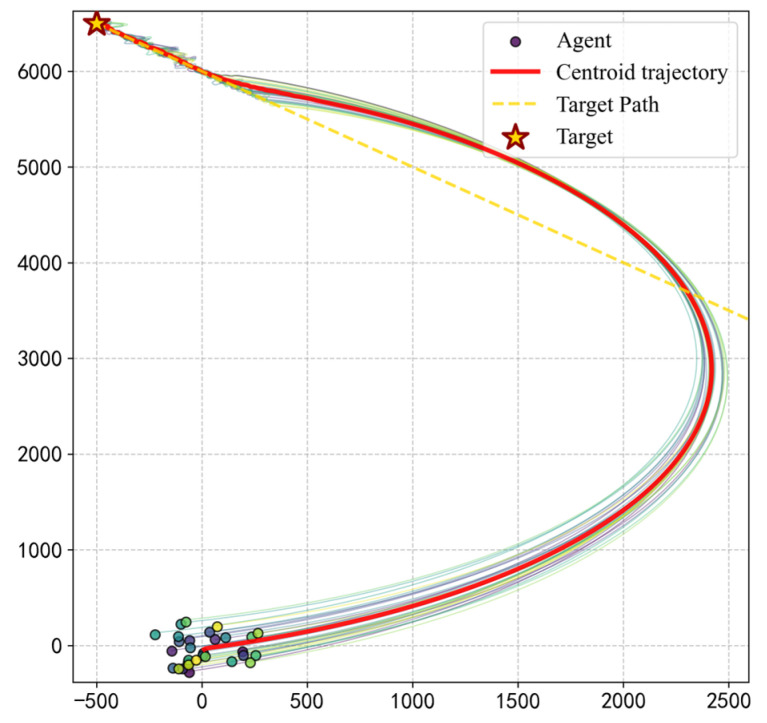
Generalization of the navigation strategy to a dynamic target tracking task. The group autonomously adjusts its heading to track a moving goal, maintaining a stable queue morphology despite the continuous shift in the relative target orientation.

As illustrated in Figure 20, the group centroid trajectory demonstrates a smooth curve that effectively tracks the moving target path. Despite the target’s continuous displacement, the individuals autonomously adjust their headings to maintain an optimal tracking angle.

**Figure 21:**
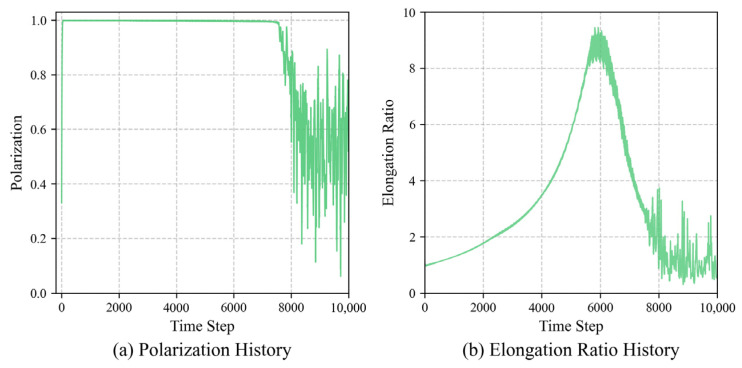
Stability of collective metrics during dynamic pursuit. Polarization and elongation remain high and consistent throughout the tracking process, demonstrating that the learned rules are robust navigation principles rather than coordinate-based mappings.

The temporal evolution of collective metrics in Figure 21 reveals the underlying stability: The group maintains a polarization $O_{p}\approx 1$ throughout the majority of the tracking process. Notably, the elongation ratio climbs to a peak of approximately 9.45 during the steady-state pursuit phase.

These results demonstrate that the learned microscopic decision rules are not merely coordinate-based mappings but represent a robust navigation principle. The model’s ability to maintain high-quality queues under dynamic constraints provides strong evidence for its applicability to complex biological and robotic scenarios.

#### 3.5.3. Generalization to Coordinate Independence

To rigorously address whether the learned strategies are biased by the specific training geometry (i.e., the target placed at the diagonal coordinates (3000,3000), we conducted coordinated independence generalization tests by rotating the target position while maintaining a constant Euclidean distance (≈4242). We placed the target at two orthogonal locations: (0,4242) and (4242,0), representing navigation along the major coordinate axes.

**Figure 22:**
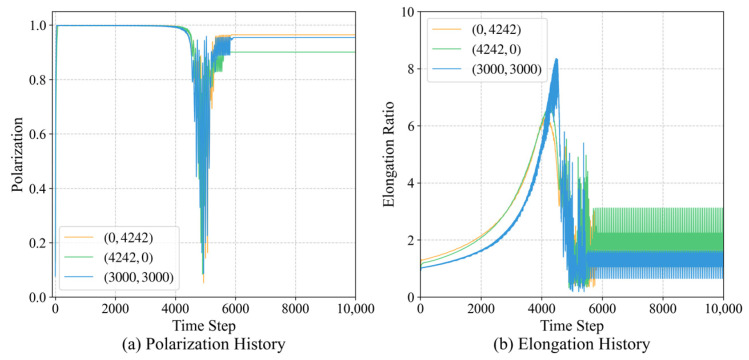
Generalization of the learned model to different target orientations. Temporal evolution of the polarization and elongation, showing that the group maintains high morphological stability regardless of the target’s absolute angular position. These results confirm the coordinate independence and rotational invariance of the learned decision-making rules.

As shown in Figure 22, the group, utilizing the *H*-matrix trained exclusively on the (3000,3000) task, successfully navigated to the new target positions with a 100% success rate without any additional parameter updates. The collective metrics remained highly consistent across all cases: the polarization $O_{p}$ quickly converged to 1, and the elongation ratio $O_{e}$ reached a steady-state value of approximately 6.5, confirming the stable formation of longitudinal queues.

This experiment provides two critical insights. First, it demonstrates that the individuals have learned a rotationally invariant navigation principle based on the relative angles ($\theta_{neighbor}$ and $\theta_{target}$) rather than a memory of absolute spatial coordinates. Second, the consistent emergence of the target-priority mechanism across different quadrants confirms that the hierarchical arbitration rule is a generalized solution for balancing social alignment and goal-seeking. These results effectively resolve the concern regarding the specificity of the training setup and underscore the robustness of the ECM-based agency model in diverse environmental configurations.

#### 3.5.4. Scalability to Larger Groups

To evaluate the scalability of the proposed model, we extended the group size from the initial N=30 to larger groups of N=100 and N=150.

**Figure 23:**
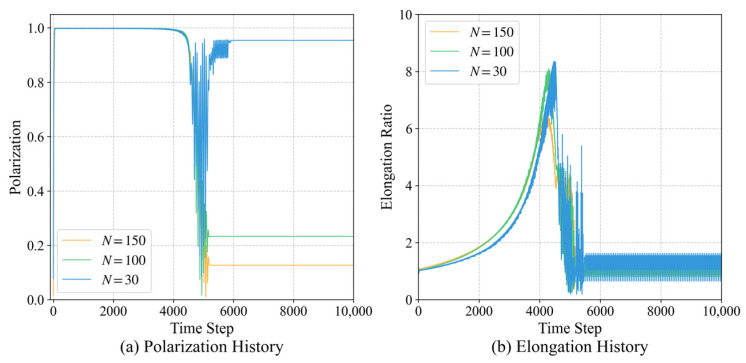
Scalability of the PS model across different group sizes (N=30,100,150). The consistency of polarization and elongation ratios indicates that the microscopic interaction rules are scale-invariant and can coordinate larger groups without further training.

As shown in Figure 23, the collective metrics remain remarkably consistent across different group sizes. The polarization $O_{p}$ consistently converges to 1, and the stable elongation ratio $O_{e}$ shows no significant degradation as *N* increases. This indicates that the microscopic interaction rules learned via ECM are scale-invariant, allowing the target-priority logic to effectively coordinate groups of varying numbers without requiring additional parameter tuning.

### 3.6. Minimal Cognitive Load and Biological Rationality

A distinctive feature of this model is the adoption of a minimalist state space consisting of only nine discrete states. While this design significantly simplifies computation by ignoring distance information, it also raises a critical theoretical question: does this simplification result in the loss of environmental information essential for navigation? Based on the experimental results, we propose and defend the concept of Minimal Cognitive Load.

Our research demonstrates that the emergence of highly ordered longitudinal queue structures does not depend on an individual’s precise distance measurements to neighbors or targets, nor does it require high-resolution continuous state perception. On the contrary, coarse-grained directional classification (i.e., simple left/center/right discrete perception) is sufficient to support complex long-distance collaborative navigation. This proves that in collective motion, the dependence of macroscopic order on microscopic information is highly compressible.

This minimalist state space design is not only computationally efficient but also possesses profound biological plausibility. Many social organisms in nature (such as arthropods like army ants and locusts) are constrained by tiny brain volumes and limited neural computing power [49,50], often making them incapable of processing complex geometric calculations or high-precision distance perception. They rely precisely on similar simple local cues (such as optic flow direction or antennal contact) to achieve efficient group synchronization [51,52]. Therefore, this model successfully demonstrates that complex spatiotemporal ordered behaviors (such as queues) can robustly emerge from minimal cognitive mechanisms consistent with the characteristics of lower organisms. This provides compelling computational evidence for understanding the low-cost navigation strategies of biological groups.

## 4. Conclusions

This study proposes an interpretable computational model of collective behavior based on Projective Simulation (PS) and Episodic Compositional Memory (ECM), aiming to reveal the microscopic mechanisms behind the emergence of efficient queue structures in long-distance navigation tasks. Unlike traditional black-box deep reinforcement learning models, this model leverages the transparent network structure of ECM, enabling us to directly parse the decision logic of individuals and establish a clear mapping from microscopic individual strategies to macroscopic collective behavior. Our key findings are summarized as follows:

**Queue Emergence and Multi-stage Evolution:** Simulation results demonstrate that under a shared-policy mode, the group can self-organize into highly ordered and slender longitudinal queue structures. The formation of this structure undergoes three distinct stages: rapid alignment, steady-state navigation, and target adjustment. Longitudinal queues effectively reduce the risk of lateral collisions and significantly enhance the navigation efficiency and stability of the group in long-distance tasks.

**Interpretable Microscopic Decision Mechanisms:** Through visual analysis of the trained ECM probability matrix, we revealed the core decision rules driving queue emergence. The study found that individuals learned a target-priority mechanism: when local neighbor alignment requirements conflict with global target navigation requirements, individuals prioritize actions pointing toward the target with high certainty. This mechanism breaks the symmetry of local interactions, providing the group with a continuous directional driving force and preventing it from falling into locally optimal aggregation states.

**Non-monotonous Effects of Action Granularity and the Intermediate Complexity Hypothesis:** Parametric analysis indicates that the discretization granularity of the action space is a key factor determining group behavior patterns, showing a significant non-monotonous relationship. Coarse granularity (e.g., Δθ=7°) results in rigid and oscillatory motion, failing to form compact structures, whereas excessively fine granularity (e.g., Δθ=1°) triggers policy noise due to the explosion of the search space, disrupting group consistency. Only at a moderate granularity (e.g., Δθ=2°) can individuals achieve the optimal balance between control precision and learning efficiency. This finding strongly supports the intermediate complexity hypothesis in swarm intelligence: overly simple individual capabilities lead to rigid collective behavior, while overly fine capabilities lead to the curse of dimensionality in the learning space; only moderate individual complexity allows for the emergence of optimal collective intelligence. This conclusion provides critical theoretical guidance for designing efficient and robust swarm systems—specifically, avoiding over-design that unnecessarily pursues high precision in individuals.

This paper bridges the gap between biological observation and computational interpretability by demonstrating that sophisticated collective order—specifically longitudinal queuing—can emerge from minimalist cognitive architectures. Our work moves beyond mere behavioral replication to uncover the underlying cognitive constraints and arbitration rules. We show that high-level navigation can be sustained by discrete, low-precision perceptions, a finding that significantly lowers the requirements for biomimetic hardware while enhancing the transparency of autonomous swarm control.

A key conceptual finding is the nature of the learning process implemented through the collective *H*-matrix. Since all agents share and update a common experience repository, this reinforcement process mirrors population-level evolution more closely than isolated individual learning. The *H*-matrix serves as a species-wide memory, refining navigation instincts across generations. This framework aligns with the biological reality of swarm-living species, where efficient coordination rules—such as the target-priority mechanism identified here—are likely the result of long-term evolutionary selection for survival and efficiency rather than high-level individual cognition. Our model thus illustrates how complex macroscopic order arises from minimal individual complexity.

In summary, this research offers an interpretable cognitive perspective on self-organizing behavior through explicit rule extraction. By revealing how simple hierarchical priorities resolve directional conflicts, we provide a robust theoretical foundation for biological navigation and a practical optimization basis for swarm robotic algorithms.

In future work, we aim to extend this framework to more complex 3D environments and multi-target scenarios and to explore the impact of group heterogeneity (e.g., different individuals with different sensing capabilities) on the robustness of collective decision-making. Crucially, we plan to validate the proposed model using physical swarm robotic platforms (e.g., e-puck or Kilobots) to bridge the gap between simulation and reality. This will provide deeper insights into the evolutionary advantages of longitudinal queuing in nature.

## Figures and Tables

**Table 1:** Simulation environment and model parameter settings.

Parameter	Symbol	Value
Initial Distribution Radius	-	300
Target Position	$P_{target}$	(3000, 3000)
Group Size	*N*	30
Individual Speed	*-*	1
Topological Neighbors	-	6
Action Space Size	*m*	15
Steering Unit	Δθ	2
Independent Runs	-	30
Training Episodes	-	5000
Max Time Steps	-	10,000
Glow Parameter	η	0.1
Reward Coeff. (Distance)	$\lambda_{d}$	0.5
Reward Coeff. (Orientation)	$\lambda_{r}$	0.5

**Table 2:** Comprehensive performance comparison between the PS model and the DQN model.

Metric	PS Model (Proposed)	DQN Model (Deep RL)
Success Rate	100%	100%
Max Elongation	8.31	8.12
Convergence Episodes	~1000	~3000
Parameters	135	>5000
Interpretability	High (Transparent Matrix)	Low (Black-box Network)
Rule Extraction	Direct Readout	Requires Extra Algorithms

**Table 3:** Impact of action discretization granularity on collective behavioral metrics.

Number of Actions	Steering Unit	Avg. Certainty	Avg. Entropy	Avg. Polarization	Max. Elongation
5	7°	0.70	0.98	0.56	3.43
11	3°	0.78	0.82	0.82	7.51
15	2°	0.91	0.33	0.95	8.31
19	1.5°	0.82	0.59	0.60	7.32
29	1°	0.39	2.22	0.49	3.41

**Table 4:** Impact of reward weights on microscopic strategy and macroscopic behavior.

Reward Coeff. (Distance)	Reward Coeff. (Orientation)	Avg. Certainty	Avg. Entropy	Avg. Polarization	Max. Elongation
0.1	0.9	0.88	0.44	0.85	3.96
0.3	0.7	0.86	0.53	0.85	3.85
0.5	0.5	0. 91	0.33	0.95	8.31
0.7	0.3	0.87	0.44	0.87	2.37
0.9	0.1	0.74	0.82	0.46	5.21

## Data Availability

Dataset available on request from the authors.
